# Antioxidant effects of ethyl acetate extract of *Desmodium gangeticum *root on myocardial ischemia reperfusion injury in rat hearts

**DOI:** 10.1186/1749-8546-5-3

**Published:** 2010-01-22

**Authors:** Gino A Kurian, Srilalitha Suryanarayanan, Archana Raman, Jose Padikkala

**Affiliations:** 1 School of Chemical and Biotechnology, SASTRA University, Thirumalaisamudram, Thanjavur, Tamil Nadu, India; 2 SASTRA University, Thirumalaisamudram, Thanjavur, Tamil Nadu, India; 3 Department of Plant Biotechnology, Amala Cancer Research Center, Amalanagar, Trichur, Kerala, India

## Abstract

**Background:**

This study aims to evaluate the antioxidant potential of the ethyl acetate extract of *Desmodium gangeticum *root for cardioprotection from ischemia reperfusion-induced oxidative stress.

**Methods:**

The *in vitro *antioxidant potential of the extract was in terms of hydroxyl radical scavenging activity, lipid peroxide scavenging activity, nitric oxide scavenging activity and diphenylpicrylhydrazyl radical scavenging activity. The *in vivo *antioxidant potential of the extract was assessed in an isolated rat heart model.

**Results:**

Free radicals were scavenged by the extract in a concentration-dependent manner within the range of the given concentrations in all models. Administration of the ethyl acetate extract of *Desmodium gangeticum *root (100 mg per kg body weight) before global ischemia caused a significant improvement of cardiac function and a decrease in the release of lactate dehydrogenase in coronary effluent, as well as the level of malondialdehyde in myocardial tissues.

**Conclusion:**

The ethyl acetate extract of *Desmodium gangeticum *root protects the myocardium against ischemia-reperfusion-induced damage in rats. The effects of the extract may be related to the inhibition of lipid peroxidation.

## Background

Many plants contain substantial amounts of antioxidants such as vitamins C and E, carotenoids, flavonoids and tannins that can be utilized to scavenge excess free radicals from the human body [1]. The free radical scavenging potential of natural antioxidants varies among diseases and types of antioxidant [2].

Antioxidants protect the human body against free radical attacks that may cause pathological conditions such as ischemia reperfusion [3]. Ischemia reperfusion causes tissue and cell damages when blood supply returns after a period of ischemia (i.e. inadequate blood supply) [4]. The onset of reperfusion in ischemic myocardium results in the release of reactive oxygen species [5]. The extensive production of reactive oxygen species during ischemia reperfusion injury is deleterious to the endogenous antioxidant defense pool. This recovery is an effective defense mechanism during the postoperative period of a patient.

Free radical scavengers and antioxidants have cardioprotective effects in experimental ischemic reperfusion models [6]. There is growing interest natural antioxidants because of the concern over the possible carcinogenic effects of synthetic antioxidants.

*Desmodium gangeticum *(*Dayeshan Ludou*, Fabaceae family) is found in India, China, Africa and Australia. It is an important plant used in the indigenous Indian medicine [7,8]*ayurveda *to treat various conditions such as snakebite, ulcer and diabetes mellitus [9,10]. The sterols, N,N-dimethyltryptamine, their oxides and other derivatives have been isolated from aerial parts of the plant; three pterocarpinoids, gangetin, gangetinin and desmodin, are the major chemical constituents of the root [11].

The present study investigates the use of ethyl acetate extract of *Desmodium gangeticum *root to protect isolated rat hearts from oxidative stress induced by ischemia reperfusion. *In vitro *and *in vivo *antioxidant models were used to assess the antioxidant potential of the herbal extract.

## Methods

### Preparation of ethyl acetate extract of Desmodium gangeticum root

The whole plant of *Desmodium gangeticum *was authenticated by Prof James Joseph. The voucher specimen A/C no. 3908 was retained in our laboratory for future reference.

The roots were dried under shade and ground to a powder (100 g) which was extracted by ethyl acetate (60-80°C) in a Soxhlet apparatus for 72 hours. The extract was concentrated under vacuum and dried at room temperature. The brownish extract (8.8 g) was resinous. Various qualitative tests [12] were performed on the extract to confirm the chemical constituents, namely triterpenoids, tannins, phenolic compounds and glycosides. All chemicals used were of analytical grade.

### Experimental animals

Adult albino Wistar male rats (weighing 250-280 g) were obtained from King Institute of Preventive Medicine, Chennai, India. They were fed on commercial rat chow (Hindustan Lever, India) and had free access to water. Handling of the animals was approved by the Indian Ministry of Social Justices and Empowerment. The experimental protocol was approved by the institutional ethics committee.

### Heart preparation

Isolated rat heart model was prepared according to Döring [13]. The rats were anesthetized at a dosage of 40 mg per kg body weight of sodium thiopentenone. After an intravenous injection of heparin (300 units), the heart was rapidly excised via a midsternal thoracotomy and arrested in ice cold Krebs-Henseleit (KH) buffer containing 118 mM/L NaCl, 4.7 mM/L KCl, 1.2 mM/L MgSO_4_, 1.2 mM/L KH_2_PO_4_, 1.8 mM/L CaCl_2_, 25 mM/L NaHCO_3 _and 11 mM/L C_6_H_12_O_6_. The heart was attached to a Lagendorff apparatus via an aorta for retrograde perfusion with KH buffer maintained at 37°C and pH7.4 and saturated with a gas mixture of 95 ml O_2 _and 5 ml CO_2_. The coronary perfusion pressure was maintained at 80 mmHg. The left ventricular pressure developed with the ventricle filled with Krebs solution was recorded with a pressure transducer, which in turn was connected to a device amplifier and chart recorder. This left ventricular pressure was an indication of the mechanical performance of the heart. Coronary flow was measured simply by collecting the perfusate draining from the heart in a graduated cylinder for a defined time. The heart rate was measured by counting the number of contractions (obtained from the left ventricular pressure recorder) per minute.

### Experimental protocol

Rats were divided into three groups. In the normal/control group (Group 1), hearts were perfused for 90 minutes with KH buffer and used for the biochemical analysis. In the reperfusion group (Group 2), the 30-minute ischemic hearts (*n *= 6 in each subgroup) were subjected to 15 minutes of reperfusion (Subgroup 2.1), 30 minutes of reperfusion (Subgroup 2.2) or 45 minutes of reperfusion (Subgroup 2.3). All animals in the treatment group (Group 3) were pretreated orally (through a ball-tipped classic steel 15-16 gauge hypodermic needle) with *Desmodium gangeticum *at a dose of 100 mg per kg body weight for 30 days and then divided into three subgroups. In Subgroup 3.1, rat hearts (*n *= 6) were perfused for 90 minutes with KH buffer and used for the biochemical analysis. In Subgroup 3.2, rat hearts (*n *= 6) were subjected to 30 minutes of global ischemia after equilibration, followed by 30 minutes of reperfusion. In Subgroup 3.3, rat hearts (*n *= 6) were subjected to 30 minutes of global ischemia after equilibration, followed by 45 minutes of reperfusion.

### Biochemical assays

Thiobarbituric acid-reactive substances (TBARS) were measured [14] as a marker of lipid peroxidation. The endogenous antioxidants, superoxide dismutases (SOD) Cu-Zn SOD and Mn SOD [15,16], catalase [17] and glutathione peroxidase [18] were estimated in a UV-1601 Shimadzu spectrophotometer (Shimadzu, USA). Protein concentration was measured with Folin phenol reagent according to Lowry *et al *[19].

### In vitro antioxidant activity

#### Determination of superoxide radical scavenging activity

Superoxide scavenging was determined by the nitroblue tetrazolium reduction method [20]. The reaction mixture consisted of ethylenediaminetetraacetic acid (EDTA; 6 μM), sodium cyanide (3 μg), riboflavin (2 μM), nitroblue tetrazolium (50 μM), various concentrations of *Desmodium gangeticum *extracts (5-50 μg/ml) and phosphate buffer (67 mM, pH7.8) in a final volume of 3 ml. The tubes were uniformly illuminated with an incandescent visible light for 15 minutes, and the optical density was measured at 530 nm before and after the illumination. The percentage inhibition of superoxide generation was evaluated by comparing the absorbance values of the control and experimental tubes.

#### Determination of hydroxyl radical scavenging activity

The scavenging capacity for hydroxyl radical was measured according to a modified method of Halliwell *et al*. [21]. Stock solutions of EDTA (1 mM), FeCl_3 _(10 mM), ascorbic acid (1 mM), H_2_O_2 _(10 mM) and deoxyribose (10 mM) were prepared in distilled deionized water. The assay was performed by adding 0.1 ml EDTA, 0.01 ml of FeCl_3_, 0.1 ml of H_2_O_2_, 0.36 ml of deoxyribose, 1.0 ml of *Desmodium gangeticum *extract (10-100 μg/ml) dissolved in distilled water, 0.33 ml of phosphate buffer (50 mM, pH7.4) and 0.1 ml of ascorbic acid in sequence. The mixture was then incubated at 37°C for 1 hour. A 1.0 ml portion of the incubated mixture was mixed with 1.0 ml of 10 g/100 g TCA and 1.0 ml of 0.5 g/100 g TBA (in 0.025 M NaOH containing 0.025 g/100 g TBA) to develop the pink chromogen measured at 532 nm. The hydroxyl radical scavenging activity of the extract is reported as percentage inhibition of deoxyribose degradation.

#### Lipid peroxide scavenging activity

A 5 ml reaction mixture containing rat liver homogenate (0.1 ml, 25 g/100 ml) in Tris-HCl buffer (40 mM, pH7.0), KCl (30 mM), ferrous iron (0.16 mM) and ascorbic acid (0.06 mM) was incubated for 1 hour at 37°C in the presence or absence of *Desmodium gangeticum *extract (20-180 μg/ml). The lipid peroxidation was measured by TBARS formation [14]. Of this incubation mixture, 0.4 ml was treated with sodium dodecyl sulphate (8.1 g/100 ml, 0.2 ml), TBA (0.8 g/100 g, 1.5 ml) and acetic acid (20 ml/100 ml, 1.5 ml, pH3.5). The total volume was then made up to 4 ml by adding distilled water and kept in a water bath at 100°C for 1 hour. After cooling, 1 ml of distilled water and 5 ml of a mixture of *n*-butanol and pyridine (15:1 v/v) was added. The mixture was centrifuged at 5000 × *g *for10 minutes and remixed. The absorbance of the organic layer was measured at 532 nm. The percentage inhibition of lipid peroxidation was determined by comparing results of the test compounds with those of controls and tubes not treated with the extracts.

#### Diphenylpicrylhydrazyl radical scavenging activity

The free radical scavenging activity of the *Desmodium gangeticum *extract and butylated hydroxyl toluene was measured with the stable radical diphenylpicrylhydrazyl (DPPH) [22] in terms of hydrogen-donating or radical-scavenging activity. A 0.1 mM solution of DPPH in ethanol was prepared, and 1.0 ml of this solution was added to 3.0 ml of extract solution in water at different concentrations (10-100 μg/ml). After 30 minutes, the absorbance was measured at 517 nm. Lower absorbance of the reaction mixture indicates higher free radical scavenging activity. The antioxidant activity of the extract was expressed as IC_50_, which was defined as the concentration (in μg/ml) of extract that inhibits the formation of DPPH radicals by 50%.

#### Nitric oxide scavenging

Sodium nitroprusside in aqueous solution at physiological pH spontaneously generates nitric oxide (NO), which interacts with oxygen to produce nitrite ions that can be estimated by use of Griess reagent [23,24]. Scavengers of NO compete with oxygen, leading to reduced production of NO. Sodium nitroprusside (5 mM) in phosphate-buffered saline was mixed with 3.0 ml of various concentrations (10-320 μg/ml) of *Desmodium gangeticum *extract dissolved and incubated at 25°C for 150 minutes. The samples were then reacted with Greiss reagent (1 g/100 ml sulphanilamide, 2 ml/100 ml H_3_PO_4_, and 0.1 g/100 ml napthylethylenediamine dihydrochloride). The absorbance of the chromophore formed during the diazotization of nitrite with sulphanilamide and subsequent coupling with napthylethylenediamine was read at 546 nm and referred to the absorbance of standard solutions of potassium nitrite also treated with Griess reagent.

### Gas chromatography-mass spectrometry (GC-MS) analysis

All GC-MS analyses were conducted with a PerkinElmer Clarus 500 gas chromatograph (Perkin Elmer, USA). The chromatographic conditions were as follows. Elite-1 (100 g/100 ml dimethylpolysiloxane) column was used. Helium was used as the carrier gas with a flow rate of 1 ml per minute. *Desmodium gangeticum *aqueous root extract (1 ml) was injected into the system in splitless mode at 250°C. The column oven temperature was maintained at 110°C for 2 minutes, then programmed at 75°C to 200°C for 1 minute and increased to 280°C by sequential increment of 5°C per minute.

### Statistical analysis

All data are presented as mean ± SD. Results were analyzed by one-way analysis of variance with SPSS software 12.00 (IBM, USA), followed by Duncan's multiple range test. *P *< 0.05 was considered statistically significant. Linear regression analysis was used to calculate IC_50 _values.

## Results

Hemodynamic changes occurred during ischemia reperfusion of the isolated rat heart. Reperfusing the ischemic heart with KH buffer did not recover the mean arterial pressure and heart rate in the early reperfusion stage of the experiment. Because heart rate and left ventricular developed pressure may recover to varying degrees, the rate pressure product was calculated by multiplying the heart rate by the left ventricular developed pressure and is presented as a reliable left ventricular function parameter for the isolated heart (Table 1). No significant difference was noted between the experimental groups for rate pressure product at the end of the 30-minute adaptation period before starting treatments and global ischemia. During the 30-minute global ischemia, there was a reduction in rate pressure product to zero, which started to recover gradually by continued reperfusion. Pretreatment with *Desmodium gangeticum *increased the recovery of the rate pressure product in the drug group (60% of basal value) compared with the reperfusion group (35% of basal value) (Table 1).

**Table 1:** Hemodynamic characteristics of rat hearts subjected to ischemia reperfusion

Group	Left ventricular developed pressure (mmHg)	Coronary flow (ml/min)	Heart rate (beats/min)	Rate pressure product ×10^3 ^(mmHg·beats/min)	Mean arterial pressure (mmHg)
**Normal control**					
1	99.21 ± 4.1	9.1 ± 1.24	340 ± 16.1	33.46 ± 4.3	121 ± 7
**Ischemia reperfusion control**					
2.1	50.43 ± 4.0*	9.0 ± 0.19	255 ± 17.2*	12.14 ± 4.2*	97 ± 6*
2.2	52.86 ± 4.3*	9.0 ± 1.10	232 ± 18.3*	11.43 ± 5.2*	96 ± 7*
2.3	40.26 ± 4.3*	9.1 ± 1.02	235 ± 30.5*	9.55 ± 7.4*	113 ± 8
**Drug treated**					
3.1	92.97 ± 4.9	9.2 ± 1.10	338 ± 27.8	31.24 ± 4.3	114 ± 7
3.2	75.21 ± 4.2*	9.1 ± 0.95	321 ± 30.2	22.22 ± 5.6*	104 ± 5*
3.3	84.70 ± 4.2	9.3 ± 1.05	320 ± 30.1	24.94 ± 7.4*	103 ± 6*

Values are mean ± SD in each group (*n *= 6). *P < 0.05, compared with control.

Gas chromatography-mass spectrometry analysis resulted in the identification of 38 compounds (Additional file 1). Major (71%) comprised *n*-hexadecanoic acid, octadecanoic acid, 1,2-benzenedicarboxylic acid, diisooctyl ester, phenol, 2,5-bis(1,1-dimethyl ethyl)-, 9-octadecenoic acid(z)-methyl ester, 2,4-bis(1-phenylethyl)phenol. Minor compounds such as cyclohexane, isocyanato azulene, 1,4-dimethyl-7-(1-methyl ethyl)-, 1-tridecanol, didodecyl phthalate, hexadecanoic acid methyl ester, 1,2-benzenedicarboxylic acid, butyloctyl ester, 1-hexadecanol and oleic acid were also identified.

Several concentrations ranging from 2 to 1000 μg/ml of ethyl acetate extract of *Desmodium gangeticum *were tested for their antioxidant activity in various *in vitro *models (Table 2). Free radicals were scavenged by the test compounds in a concentration-dependent manner within the given range of concentrations in all the models. The half maximum inhibitory concentration (IC_50_) in the DPPH, superoxide scavenging activity, hydroxide scavenging activity, nitric oxide scavenging activity and lipid peroxidation models were 36.3, 55.3, 43.7, 39.4 and 248 μg/ml respectively (Table 2 &3).

**Table 2:** Free radical scavenging activities of *Desmodium gangeticum *extract

Extract concentration (μg/ml)	Inhibition (%)			
	DPPH	Nitric oxide	Superoxide	Hydroxyl radical
1000	89.25 ± 2.11	87.21 ± 3.11	92.31 ± 2.63	81.27 ± 3.82
500	86.49 ± 3.46	82.28 ± 5.23	87.66 ± 3.51	78.63 ± 4.62
250	81.67 ± 2.34	77.55 ± 3.45	79.41 ± 3.65	74.41 ± 4.43
125	75.22 ± 3.74	70.39 ± 4.84	67.51 ± 2.78	65.52 ± 2.76
62	46.83 ± 2.28	46.63 ± 5.28	61.39 ± 3.51	51.62 ± 3.52
32	32.57 ± 3.38	38.68 ± 4.38	50.47 ± 2.54	30.61 ± 2.31
16	4.48 ± 2.55	19.25 ± 3.27	39.78 ± 2.89	21.42 ± 1.62
10	2.21 ± 1.52	7.52 ± 1.32	29.37 ± 1.12	4.21 ± 0.52
7	1.02 ± 0.74	4.33 ± 0.50	19.67 ± 1.44	3.34 ± 1.25
5	0.10 ± 0.03	1.31 ± 0.10	7.21 ± 1.05	1.23 ± 0.33
Ascorbic acid (100 μg)	95.11 ± 4.22	85.34 ± 4.11	87.32 ± 5.87	94.44 ± 4.71
Butylated hydroxytoluene (20 μg)	92.27 ± 3.31	NT	NT	NT
Curcumin	NT	91.7 ± 3.11	NT	NT
IC_50_	36.3 ± 1.47	39.4 ± 2.33	55.3 ± 1.29	43.7 ± 2.43

Values are mean ± SD of three replicates. NT: Not tested.

**Table 3:** Effects of ethyl acetate root extract of *Desmodium gangeticum *on ferrous sulphate-induced lipid peroxidation in rat liver homogenate

Extract concentration (μg/ml)	TBARS (nmol/mg protein)^a^	Inhibition (%) ^a^
Control	2.32 ± 0.27	
1000	0.1 ± 0.02	96.34 ± 2.7
800	0.38 ± 0.04	83.75 ± 2.6
600	0.55 ± 0.12	76.21 ± 2.1
400	0.87 ± 0.14	62.36 ± 2.5
200	1.00 ± 0.23	58.75 ± 2.4
Tocopherol (10 μmol/L)	0.07 ± 0.02	97.11 ± 3.5

^a ^Mean ± SD of *n *= 6

The *in vivo *antioxidant effect of the extract was determined by administering the rats with *Desmodium gangeticum *orally for 30 days and then sacrificing them for reperfusion-induced ischemic injury. Lipid peroxidation in drug treated rat hearts were reduced as compared to ischemia reperfusion control hearts. Similarly antioxidant enzymes also recovered significantly in drug treated rat hearts (Table 4). These observations in the present study suggest a potent *in vivo *antioxidant capacity for *Desmodium gangeticum *against revascularization injury.

**Table 4:** Effects of ethyl acetate root extract of *Desmodium gangeticum *on TBARS, catalase, superoxide dismutase (SOD), and glutathione peroxidase (GPx) in the tissue homogenate of isolated rat hearts

Group	TBARS (μM/g wet tissue)	Catalase (μM of H_2_O_2 _consumed/min/g protein)	SOD (U/mg protein)^#^		GPx (μg of GSH consumed/min/g protein)
			Mn SOD	Cu-Zn SOD	
**Normal control**					
1	6.1 ± 0.2	7617 ± 441	8.1 ± 0.62	50.2 ± 4.1	1859 ± 181
**Ischemia reperfusion control**					
2.1	7.9 ± 0.6*	4 087 ± 246*	5.1 ± 0.52*	30.3 ± 3.5*	1228 ± 142*
2.2	7.5 ± 0.5*	5176 ± 372*	6.1 ± 0.54*	34.1 ± 3.2*	1117 ± 114*
2.3	7.1 ± 0.5*	5208 ± 316*	5.6 ± 0.57*	33.8 ± 3.8*	1216 ± 116*
**Drug treated**					
3.1	5.9 ± 0.3	7856 ± 447	8.0 ± 0.71	50.1 ± 4.3	1855 ± 178
3.2	5.9 ± 0.3	7573 ± 433	8.0 ± 0.78	51.0 ± 4.9	1804 ± 183
3.3	4.8 ± 0.2*	6176 ± 455*	7.1 ± 0.62*	44.3 ± 4.1	1572 ± 176*

^#^SOD unit: One unit is defined as the enzyme concentration required to inhibit the optical density (at 560 nm) produced by 50% of chromogen 50% in 1 minute.

Values are mean ± SD in each group (*n *= 6). Significantly differing values (from normal control group) are marked with an asterisk (*P *< 0.05).

Cardiac enzymes like CK, LDH, SGOT and SGPT in the tissue homogenate were significantly high in ischemia reperfusion control rats (Table 5). However administration of the DG root extract improved the level of these enzymes and thereby mediates myocardial protection.

**Table 5:** Activities of creatine kinase, lactate dehydrogenase, SGOT, and SGPT in the tissue homogenate of isolated rat hearts

Group	Creatine kinase (μmol phosphorous liberated/min/mg protein)	Lactate dehydrogenase (nmol pyruvate liberated/min/mg protein)	SGOT (nanomol pyruvate generated/min/mg protein)	SGPT (nanomol pyruvate generated/min/mg protein)
**Normal control**				
1	16.1 ± 1.4	104.4 ± 8.7	35.3 ± 4.1	26.1 ± 2.5
**Ischemia reperfusion control**				
2.1	9.2 ± 0.8*	62.4 ± 4.6*	19.8 ± 1.1*	14.6 ± 1.2*
2.2	10.1 ± 2.7*	59.5 ± 7.2*	17.1 ± 3.8*	13.3 ± 2.2*
2.3	10.4 ± 2.2*	57.1 ± 7.8*	16.6 ± 3.1*	14.9 ± 2.7*
**Drug treated**				
3.1	15.55 ± 2.1	85.6 ± 8.1*	26.5 ± 1.3*	23.5 ± 2.1
3.2	15.56 ± 1.6	90.8 ± 6.5*	29.8 ± 2.8*	26.6 ± 1.9
3.3	15.84 ± 1.8	93.8 ± 6.9	30.5 ± 4.3	27.5 ± 2.8

Values are mean ± SD in each group (*n *= 6).

Values that differ significantly from normal control group are marked with an asterisk (*P *< 0.01).

## Discussion

Previous studies on the use of medicinal plants to treat cardiac disorders suggested that methanol extract of *Desmodium gangeticum *root renders cardioprotection from isoproterenol-induced myocardial infarction in rats [25,26]. The preventive effects of ethyl acetate extract of *Desmodium gangeticum *root were shown in terms of cardiac marker enzymes and antioxidants in ischemic reperfused rat hearts. We found that ethyl acetate extract of *Desmodium gangeticum *root induces myocardial protection against ischemia reperfusion injury in isolated rat hearts, as indicated by the improved recovery of cardiac function, reduction in cardiac enzyme release in the perfusate and reduction of tissue necrosis.

The functional recovery of myocardium from ischemia reperfusion-induced assault was observed through the changes in hemodynamic parameters (Table 1). Significant recovery of left ventricular developed pressure in drug-treated rat heart suggested the physiological recovery of heart from ischemia reperfusion injury. Similarly, improvement of rate pressure product and mean arterial pressure in ethyl acetate-treated rat heart explained the recovered ionic balance for the normal physiological functions of hearts.

**Figure 1:**
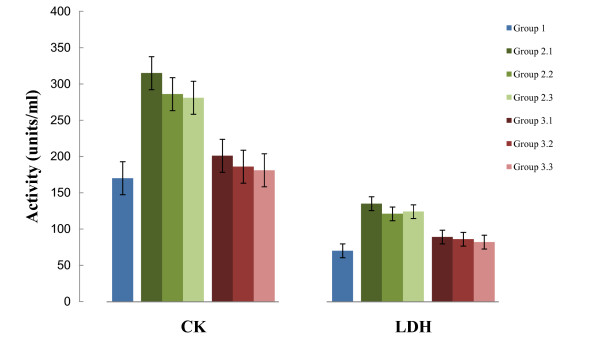
**Activities of creatine kinase and lactate dehydrogenase in the perfusate of isolated rat hearts**. Group 1: normal control; Group 2.1, 2.2, 2.3: ischemic reperfusion control; Group 3.1, 3.2, 3.3: drug pretreated and subjected to ischemic reperfusion. Values are mean ± SD in each group (*n *= 6).

The cardiac damage due to ischemia reperfusion was monitored by the presence of cardiac marker enzymes in the cardiac perfusate and the level of these enzymes in myocardium. The presence of lactate dehydrogenase and creatine kinase in coronary perfusate of isolated rat heart indicated myocardial necrosis [27]. In this study, however, the levels of these enzymes in perfusate were limited (Figure 1) and a subsequently increased level was found in the myocardial tissue of rat hearts treated with ethyl acetate extract (Table 5).

The superoxide anion scavenging activity of ethyl acetate extract of *Desmodium gangeticum *root increased markedly with the increase of concentrations (Table 2), and the IC_50 _of the extract was 55.3 μg/ml. The *Desmodium gangeticum *extract exhibited concentration-dependent scavenging activities against hydroxyl radicals generated in a Fenton reaction system, and the IC_50 _of the extract was 43.7 μg/ml (Table 2). NO is known to be involved in inflammation, cancer and other pathological conditions [28]. The *Desmodium gangeticum *extract moderately inhibited NO in a dose-dependent manner (Table 2), and the IC_50 _was 39.4 μg/ml. The *Desmodium gangeticum *extract inhibited FeSO_4_-induced lipid peroxidation in rat liver in a dose-dependent manner. The DPPH method is a simple, rapid, and convenient method independent of sample polarity for screening of many samples for radical scavenging activity [29]. The extract IC_50 _value as measured by the DPPH method was 36.3 μg/ml.

*In vivo *antioxidant potential of ethyl acetate extract of *Desmodium gangeticum *root was determined in isolated rat hearts. A massive release of reactive oxygen species was identified as one of the main causative factors for myocardial ischemia reperfusion injury [6]. Xanthine dehydrogenase, which normally utilizes NADH as an electron acceptor, is converted under the conditions of ischemia/reperfusion into xanthine oxidase, which uses oxygen as a substrate [30]. Similarly, NADPH oxidase and mitochondrial electron transport chain complexes were reported as the other sources of free radicals [6]. In the present study, increased myocardial TBARS indicated oxidative stress induced by myocardial ischemia reperfusion injury. However, administration of *Desmodium gangeticum *extract not only reduced TBARS in myocardium but also enhanced the recovery of antioxidant enzymes from the assault of ischemia reperfusion injury (Table 4).

## Conclusion

The ethyl acetate extract of *Desmodium gangeticum *root protects the myocardium against ischemia-reperfusion-induced damage in rats. The effects of the extract may be related to the inhibition of lipid peroxidation.

## Abbreviations

DG: *Desmodium gangeticum*; BHA: Butylated hydroxyanisole; BHT: Butylated hydroxytoluene; IRI: Ischemia reperfusion injury; ROS: Reactive oxygen species; KH: Krebs - Henseleit buffer; TBARS: Thiobarbituric acid reactive substances; SOD: Superoxide dismutase; GPx: Glutathione peroxidase; NBT: Nitroblue tetrazolium; DPPH: diphenylpicrylhydrazyl; MAP: Mean arterial pressure; HR: Heart rate; LVDP: Left ventricular developed pressure; RPP: Rate pressure product

## Competing interests

The authors declare that they have no competing interests.

## Authors' contributions

GAK designed the study, performed the experiment, interpreted the data and prepared the manuscript. SS and AR performed the experiment and revised the manuscript. JP designed the study, interpreted the data and revised the manuscript. All authors read and approved the final version of the manuscript.

## Supplementary Material

Additional file 1 Chemical composition of ethyl acetate extract of *Desmodium gangeticum *root by gas chromatography-mass spectrometry Click here for file
