# Supplementary material for: Antioxidant effects of ethyl acetate extract of Desmodium gangeticum root on myocardial ischemia reperfusion injury in rat hearts
Source: Chin Med. 2010 Jan 22;5:3. doi: 10.1186/1749-8546-5-3 (PMC2831010; doi:10.1186/1749-8546-5-3)
Supplement: Additional file 1 — Chemical composition of ethyl acetate extract of Desmodium gangeticum root by gas chromatography-mass spectrometry [file 1749-8546-5-3-S1.DOC]

Additional file 1

Chemical composition of ethyl acetate extract of *Desmodium gangeticum* root by gas chromatography-mass spectrometry

| **No.** | **Retention time (s)** | **Name of the compound** | **Peak %** |
| --- | --- | --- | --- |
| 1 | 3.79 | hexanoic acid | 0.23 |
| 2 | 4.23 | cyclohexane, isocyanato | 1.05 |
| 3 | 5.72 | methyl-2-O-benzyl-d-arabinofuranoside | 0.19 |
| 4 | 6.49 | hexanediamide, *n,n*’-dibenzoyloxy- | 0.52 |
| 5 | 6.88 | 1-O-acetyl-exo-2,3-O-ethylidene-β-d-erythrofuranose | 0.38 |
| 6 | 6.93 | benzoic acid, 2-butoxy-methyl ester | 0.94 |
| 7 | 7.00 | cyclohexane, (3-methlpentyl) | 0.25 |
| 8 | 7.24 | cyclohexane, 1,1’-(1,2-dimethyl-1,2-ethanediyl)bis- | 0.39 |
| 9 | 7.35 | cyclohexane, isothiocyanato | 0.52 |
| 10 | 7.63 | cyclohexane, hexyl | 0.50 |
| 11 | 7.77 | nonanoic acid | 0.24 |
| 12 | 8.21 | 1,3-benzodioxole,5-(2-propenyl)- | 0.37 |
| 13 | 9.40 | 5-eicosene (e) | 0.35 |
| 14 | 9.52 | 1-undecanol | 0.98 |
| 15 | 9.64 | decane 2,3,5,8-tetramethyl- | 0.63 |
| 16 | 10.40 | cyclohexane, undecyl- | 0.34 |
| 17 | 10.78 | benzene,1-(1,5-dimethyl-4-hexyenyl)4 methyl- | 0.94 |
| 18 | 11.93 | 1,4-benzenedicarboxylic acid methyl ester | 0.80 |
| 19 | 12.16 | oxalic acid, allyl pentadecyl ester | 0.45 |
| 20 | 13.03 | 6-tridecanone | 0.59 |
| 21 | 13.67 | benzoic acid, 2-ethylhexyl ester | 0.40 |
| 22 | 13.92 | azulene, 1,4-dimethyl-7-(1-methyl ethyl)- | 2.46 |
| 23 | 14.31 | phenol, 2,5-bis(1,1-dimethyl ethyl)- | 4.70 |
| 24 | 14.81 | 1-tridecanol | 1.60 |
| 25 | 15.75 | didodecyl phthalate | 1.29 |
| 26 | 16.67 | hexadecanoic acid methyl ester | 1.32 |
| 27 | 17.16 | 1,2-benzenedicarboxylic acid, butyl octyl ester | 1.48 |
| 28 | 17.39 | *n*-hexadecanoic acid | 34.68 |
| 29 | 17.84 | 1-hexadecanol | 1.19 |
| 30 | 19.41 | 9-octadecenoic acid(z)-methyl ester | 3.68 |
| 31 | 19.80 | cyclopentaneundecanoic acid, methyl ester | 0.91 |
| 32 | 20.50 | octadecanoic acid | 16.34 |
| 33 | 20.96 | oleic acid | 1.46 |
| 34 | 21.35 | 2-tridecenal | 2.04 |
| 35 | 22.39 | 4-trifluroacetoxypentadecane | 1.63 |
| 36 | 24.34 | 2,4-bis(1-phenylethyl)phenol | 3.89 |
| 37 | 25.17 | cyclopentaneundecanoic acid, methyl ester | 2.64 |
| 38 | 25.49 | 1,2 benzenedicarboxylic acid, diisooctyl ester | 7.61 |
